# Effects of a virtual gender swap on social and temporal decision-making

**DOI:** 10.1038/s41598-021-94869-z

**Published:** 2021-07-28

**Authors:** Elena Bolt, Jasmine T. Ho, Marte Roel Lesur, Alexander Soutschek, Philippe N. Tobler, Bigna Lenggenhager

**Affiliations:** 1 Department of Psychology, Cognitive Neuropsychology with Focus on Body, Self, and Plasticity, University of Zurich, Binzmühlestrasse 14, Box 9, 8050 Zurich, Switzerland; 2 Department of Psychology, Ludwig Maximilian University, Munich, Germany; 3 Laboratory for Social and Neural Systems Research, Department of Economics, Zurich Center for Neuroeconomics, University of Zurich, Zurich, Switzerland

**Keywords:** Neuroscience, Cognitive neuroscience, Reward, Social behaviour

## Abstract

Mounting evidence has demonstrated that embodied virtual reality, during which physical bodies are replaced with virtual surrogates, can strongly alter cognition and behavior even when the virtual body radically differs from one’s own. One particular emergent area of interest is the investigation of how virtual gender swaps can influence choice behaviors. Economic decision-making paradigms have repeatedly shown that women tend to display more prosocial sharing choices than men. To examine whether a virtual gender swap can alter gender-specific differences in prosociality, 48 men and 51 women embodied either a same- or different-gender avatar in immersive virtual reality. In a between-subjects design, we differentiated between specifically social and non-social decision-making by means of a virtually administered interpersonal and intertemporal discounting task, respectively. We hypothesized that a virtual gender swap would elicit social behaviors that stereotypically align with the gender of the avatar. To relate potential effects to changes in self-perception, we also measured implicit and explicit identification with gendered (or gender-typical) traits prior to and following the virtual experience, and used questionnaires that assessed the strength of the illusion. Contrary to our hypothesis, our results show that participants made less prosocial decisions (i.e., became more selfish) in different-gender avatars, independent of their own biological sex. Moreover, women embodying a male avatar in particular were more sensitive to temptations of immediate rewards. Lastly, the manipulation had no effects on implicit and explicit identification with gendered traits. To conclude, while we showed that a virtual gender swap indeed alters decision-making, gender-based expectancies cannot account for all the task-specific interpersonal and intertemporal changes following the virtual gender swap.

## Introduction

Embodied cognition proposes a strong reciprocal link between bodily and cognitive processes. While such a relation was initially proposed primarily for the physical body, it has increasingly been extended to the subjectively perceived body as well, which is known to be plastic and shaped by both sensory inputs and prior expectancies (e.g., Dijkermann and Lenggenhager^1^). In line with this idea, mounting evidence has demonstrated that experimentally induced temporary changes in the sense of one’s own body can alter a broad range of cognitive and affective processes, including social cognition (for a review, see Maister et al.^2^). This relation has often been investigated with multisensory stimulation paradigms, such as the rubber hand illusion^3^, and more recently also in virtual reality (VR). The precise sensory control of the virtual body, coupled with synchronous visuotactile and/or visuomotor feedback between the participant’s own body and the avatar, make VR a powerful tool to induce a strong temporary sense of embodiment, even in the presence of salient dissimilarities, such as different skin tones^4^ or gender^5,6^. It has been suggested that such alterations of embodiment induce a so-called Proteus effect, where the embodiment of an avatar elicits behaviors and cognition concomitant with attributes and stereotypes of the embodied avatar^7^. This effect is believed to result from a deindividuation process in virtual environments, during which “virtual cues take precedence over physical cues” (Yee et al.^8^, p. 3). Along these lines, virtual body swap studies have demonstrated that embodiment of avatars that visually diverge from one’s physical characteristics can exert strong influences on cognition. For example, embodiment of an elderly^9^ or dark-skinned^4^ person can reduce negative stereotypes towards such outgroup members in young or light-skinned adults, respectively. Furthermore, avatar embodiment might not only change the perception of others but also the self, as the identification with a child-like virtual body, for example, results in stronger self-association of child-like personality traits^10^, as well as in a shift towards a more child-like pitched voice^11^.

Gender swap illusions have similarly prompted users to engage in stereotype-relevant behaviors, such as conforming to gender-stereotyped language norms when embodying gendered avatars^12^ or engaging more in killing versus healing behaviors when embodying male versus female avatars^13^. In one study, female participants were less sensitive to the effects of stereotype threat (i.e., being told that women tend to perform worse than men on a working memory task they were about to complete) when embodying a male compared to a female avatar^5^. On the other hand, male participants underperformed when embodying a female avatar^6^. Another study reported that when being caressed in intimate areas by a person of the same-gender, heterosexual female and male participants’ ratings of pleasantness and erogeneity were higher during virtual embodiment of a body of the different-gender as compared to a same-gender toucher^14^. Tacikowski et al.^15^ demonstrated that a few minutes of immersion in a gender swap illusion resulted in altered implicit and explicit identification with gendered (or gender-typical) traits, particularly in a shift towards a more balanced identification with both genders, as well as concomitant decreases in gender-stereotyped beliefs about oneself. While most of these paradigms examined changes during or immediately following the illusion, a few studies have specifically assessed the longevity of cognitive alterations following virtual embodiment, and determined that some virtual embodiment manipulations induced changes that persisted for at least one week^16^. Findings such as these demonstrate that alterations in cognition following transient modifications of embodiment can potentially persist even outside the laboratory. Therefore, a more finetuned understanding of how virtual body swaps can affect cognitive affective processes will prove informative not only for basic research, but may hold also practical, consumer-level relevance for assessing potential consequences of spending extended time as an avatar in virtual environments.

One major theme of economic games surrounds the notion that humans care about and are motivated by the interests of others. As a major building block of prosocial behavior, generosity (i.e., sharing behaviors) has been incorporated into numerous economic models as a measure of social preferences^17^. It should be noted that we here use the term “generosity” specifically as a description of the willingness to incur a relative disadvantage in order to benefit someone else. A repeatedly documented source of heterogeneity in generosity has been linked to the differential gender-specific social preferences of men and women^18^. While prosocial and generous behaviors have characteristically been ascribed to women (where they are stereotypically perceived as a favorable trait), more selfish behaviors have in turn been generally characterized as a typecast male trait^19^. Several behavioral economic studies have corroborated robust gender differences in social decision-making^20^, while a recent neuropharmacological study by Soutschek et al.^21^ further substantiated such gender-specific prosocial preferences on a neural level by showing increased responses in the striatum, a component of the neural reward system, to prosociality in women.

To add to the fundamental research on the contribution of altered bodily self experiences to cognitive processes, this study aimed to alter participants’ gender identity using an immersive virtual gender swap illusion (i.e., using avatars that are different to the self in gender stereotypical physical features), and to examine the effects of this virtual gender swap on social decision-making. Examining such cognitive alterations in digital spaces not only contributes to the fundamental research on bodily self-consciousness, but further holds real-world implications, especially as an ever-increasing number of social interactions occur in digital environments. To this purpose, healthy female and male participants embodied a typified avatar representative of either the “female” or “male” gender in a virtual environment, in which they completed an interpersonal (generosity, as a measure of prosociality) and intertemporal (delay discounting) discounting task. Based on several empirical findings evincing that women tend to make more prosocial decisions^19–23^, we expected participants embodying a female avatar to make more prosocial choices than those embodying a male avatar, independent of their own biological sex. An intertemporal discounting task was chosen as a control task, since research has previously demonstrated no gender differences in intertemporal discounting, and other studies have similarly used intertemporal discounting as a control task^21^. To more directly relate potential effects of virtual embodiment to changes in self-perception, we measured implicit and explicit identification with gendered (or gender-typical) traits, as well as voice pitch before, during, and after avatar embodiment with the hypothesis that the different-gender avatar would reduce gender identification (Tacikowski et al.^15^ and alter voice pitch^11^ towards its typified gender.

## Methods

### Participants

We recruited a female sample (*n* = 52) and a male sample (*n* = 52). Male and female participants were recruited in separate calls (one call specifically looking for “women”, i.e., participants that identified as female, and the other call specifically looking for “men”, i.e., participants that identified as men) via the student mailing lists of the Economics and Psychology Departments at the University of Zurich, as well as through personal contacts. While we did not specifically ask them about their biological sex, the call for participants was directed towards students with clearly defined gender identity. They were assigned to the gender-swap group (experimental groups) or no gender-swap group (control groups) by sequential counterbalancing, i.e., they were alternatingly allocated to the experimental (different-gender) or the control (same-gender) group as they signed up for the study. This resulted in two experimental groups, referred to as *Fem-Male* (female participants embodying a male avatar) and *Male-Fem* (male participants embodying a female avatar), and two control groups, referred to as *Fem-Fem* (female participants embodying a female avatar) and *Male-Male* (male participants embodying a male avatar). One female participant and four male participants had to be excluded due to technical issues, resulting in a final sample of *n* = 26 (23.5 ± 2.3 years) in Fem-Male, *n* = 25 (23.5 ± 4.7 years) in Fem-Fem, *n* = 23 (23.7 ± 3.3 years) in Male-Fem, and *n* = 25 (23.0 ± 2.7 years) in Male-Male. Inclusion criteria were good health (self-assessed and self-reported; we explicitly called for “healthy women/men” in the advertisement of the study). Compensation consisted either of course credit or a fixed payment of 25 Swiss francs. Additionally, all participants received a specific payment, which was dependent on their choices in the interpersonal and intertemporal discounting tasks (ranging from a minimum of 7.50 to a maximum of 31.50 Swiss francs). The protocol is in line with the Helsinki declaration and was approved by the Ethics Committee of the Faculty of Arts and Social Sciences of the University of Zurich (approval number 17.12.15). All participants gave their written informed consent prior to participation.

### Equipment and virtual environment

**Figure 1:**
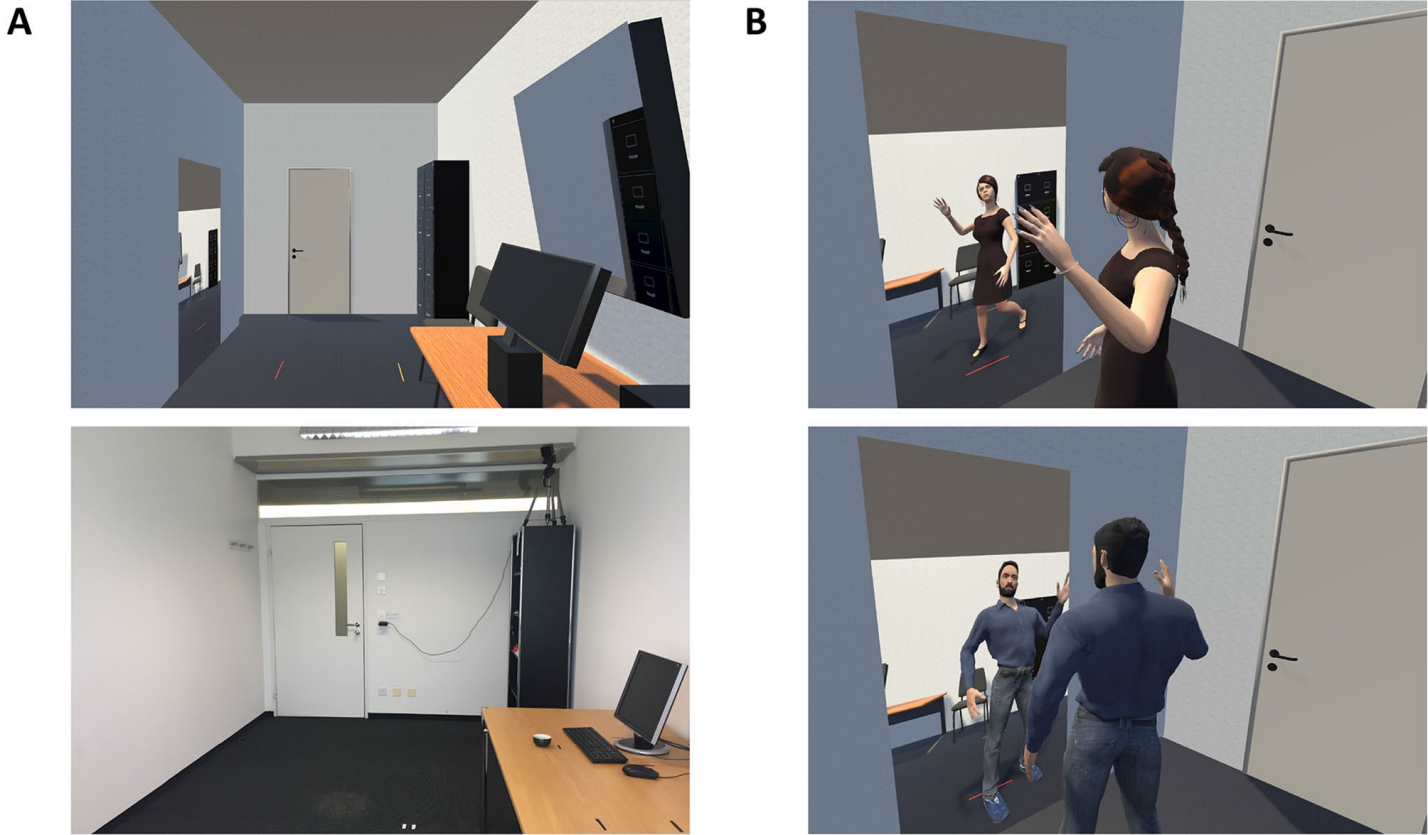
Virtual environment and avatars. (**A)** Virtual (top) and real (bottom) room in which participants moved around and the experiment took place. (**B)** Typified female (top) and male (bottom) avatar moving in front of the full body displaying mirror.

Participants wore an HTC Vive Pro (Vive™), a head-mounted display with a resolution of 1440 × 1600 pixels per eye and a diagonal field of view of 110°, four Vive Trackers respectively placed on the left and right wrists and ankles, and a Vive Controller attached to the front of the midsection. The trackers and controller served as tracking points to enable tracker-to-body-matching with the avatar. The virtual environment was implemented in the Unity 2018.2 game engine (see https://unity.com/). The Steam VR package (Steam®) was used to functionally combine the Vive kit with Unity for full body tracking. To enable inverse kinematics (in order to synchronize real movements with virtual movements), we used the Final IK VR Inverse Kinematics package, an animation tool provided by Unity RootMotion. The virtual environment was designed to simulate the real environment; therefore, the virtual room strongly resembled the actual testing room (Fig. 1A) in which the experiment took place. The main furniture consisted of a desk with a computer, which was used to complete the two discounting tasks. Since real-time mirror reflections have been shown to enhance virtual embodiment^24^, we added two mirrors to the virtual environment. One large, full body mirror was placed on the wall (Fig. 1B) and one smaller mirror displaying only the upper body was placed directly behind the computer, so that participants could see the avatar’s virtual reflections while completing the tasks on the virtual computer screen.

### Tasks and measures

#### Interpersonal and intertemporal discounting tasks

Following Soutschek et al.^21^, we adapted the tasks from Jones and Rachlin^25^ to assess generosity and delay discounting. In the interpersonal discounting task, participants chose between a generous and a selfish choice option. If they chose the generous option, they shared a reward of 15 Swiss francs (7.50 each) with a person of varying social distance, whereas the selfish option offered a varying amount (7.50 to 15.50 Swiss francs) to participants. The typically larger amount provided by the selfish option to the participant came at the expense of the other person not receiving any financial reward. Six different social distances (1, 5, 10, 20, 50 and 100) were defined beforehand in a social distance questionnaire. The scale represented the social environment of participants and ranged from 0 to 100, where 0 represented “yourself”, 100 represented a “stranger seen randomly on the street”, and 50 “someone you have seen before, but whose name you do not know”. They were then asked to name specific persons from their social environment (to whom they did not have a negative relation) for distances 1, 5, 10 and 20. In the intertemporal discounting task, participants chose between a smaller-sooner or larger-later choice option. Smaller-sooner options varied from 0 to 16 Swiss francs and were paid “today” (i.e., the day of participation). The larger-later option was 16 Swiss francs with varying temporal distances, i.e., to be paid in 1, 10, 20, 50, 90 or 180 days after their participation. To promote honest and accurate decisions according to their true preferences, participants were informed that one trial for each task would be drawn at random at the end of the experiment, and paid out according to their decision. They were also truthfully informed that the other person (either a person they named or a stranger, depending on the social distance of the drawn trial) would be paid the amount specified in the drawn trial.

#### Implicit association task (IAT)

The IAT measures the categorization speed and accuracy in conditions involving verbal or pictorial stimuli and is therefore used to assess automatic association strength between two concepts^26^. The Self-Concept IAT combines the contrast of “self” versus “other” with a contrast of interest^27^. In the current study, the contrast of interest was “female” versus “male” to which participants had to associate pictorial stimuli of female and male faces. A positive *d* score indicates faster sorting of “female” with “self” and “male” with “other”, whereas a negative *d* score indicates faster sorting of “female” with “other” and “male” with “self”. We used the *d* score as a measure of implicit self-association with a gender. The IAT was implemented in PsyToolkit^28,29^.

#### Voice recordings

Participants’ voices were recorded using a Zoom H6 Hand Recorder (Zoom Corporation, Japan) with an SGH-6 Shotgun Microphone Capsule at a sampling rate of 44.1 kHz at 24 bits. Each participant was stationed at a similar distance to the microphone, indicated by a mark on the virtual floor during the recording (as well as by a mark on the actual floor for the experimenter to confirm the appropriate location). The recording was started manually prior to beginning and upon completion of each task. Each task consisted of five repetitions of nine different made-up two syllable words that appeared on the VR headset, resulting in a total of 45 words. Each item was spelled with four letters and appeared on the screen for 1000 ms, followed by a pause of 500 ms. The applied procedure for the voice recordings is similar to those used in previous studies^11,16,30^.

#### Gender adjectives

We asked participants to rate how strongly they associate themselves with nine adjectives representing typically “feminine” or “masculine” categories^31,32^ on a visual analogue scale (VAS). “Female” terms were placed on the left VAS pole and “male” terms on the right, so that the choices on the VAS (ranging from 0 “female” to 1 “male”) could be calculated as a measure of explicit self-association with gender. The word pairs were: modest–dominant, social–egoistic, tender–rough, emotional–rational, weak–strong, soft–hard, romantic–realistic, flexible–stubborn, and anxious–unafraid.

#### SVO task

People differ in how they evaluate outcomes in relation to themselves versus others. Messick and McClintock^33^ described three distinct Social Value Orientations (SVO): prosocial, individualist, and competitor. To measure participants’ SVO, we implemented nine game items suggested in Van Lange et al.^34^ into the virtual environment. For each item, participants made a choice between three different combinations of outcomes to self and other (e.g., option A: 480 points to self and 80 points to other, B: 540 points to self and 280 points to other, C: 500 points to self and 100 points to other). Each of the three combinations corresponded to one of the three SVO. Participant SVO was determined based on if choices corresponded to the same SVO for at least six out of nine items. The SVO task was implemented in VR and participants used the Vive’s eye camera to pick their choices. Participants classified as prosocial are assumed to assign a positive value to both their own and others’ outcomes, while those classified as individualist exclusively assign weight to outcomes for themselves, and those classified as competitor prioritize the relative advantage of their own outcomes compared to those of others^35^. Our aim was to use the SVO as a baseline measure of and control measure for prosociality between the different groups.

#### Movement instructions

Audio instructions requesting participants to move around the virtual room by following predetermined points and to explore their “new” body in the mirrors were played through the Vive’s integrated earphones. The experimenter’s pre-recorded female voice instructed participants to perform a standardized set of movements, such as to wave to themselves or to alternately lift their legs in front of the mirrors. These tasks were done to call attention to the visuomotor synchrony between the virtual and physical body, and therefore to maximize the sense of embodiment of the virtual avatar (for a transcript of the audio instructions, see the supplementary materials).

#### Measure of embodiment

To assess the strength of the embodiment illusion, participants answered an Embodiment Questionnaire, which was adapted from Peck et al.^5^ Participants answered on a virtual VAS ranging from 0 (strongly disagree) to 1 (strongly agree).

Q1: I felt as if the body I saw in the virtual world might be my body.

Q2: I felt like the body in the virtual world was someone else.

Q3: I felt like the body in the mirror was my body.

Q4: I felt like I controlled the virtual body as if it were my own body.

Q5: I felt like I was embodying a male/female body.

#### Debriefing questions

At the end of the experiment, participants answered the following debriefing questions, again on a virtual VAS ranging from 0 (strongly disagree) to 1 (strongly agree).

D1: I felt like my decisions on the tasks were influenced by the virtual body.

D2: I felt like the virtual body looked similar to my own body.

D3: I liked the virtual body’s appearance.

### Procedure

**Figure 2:**
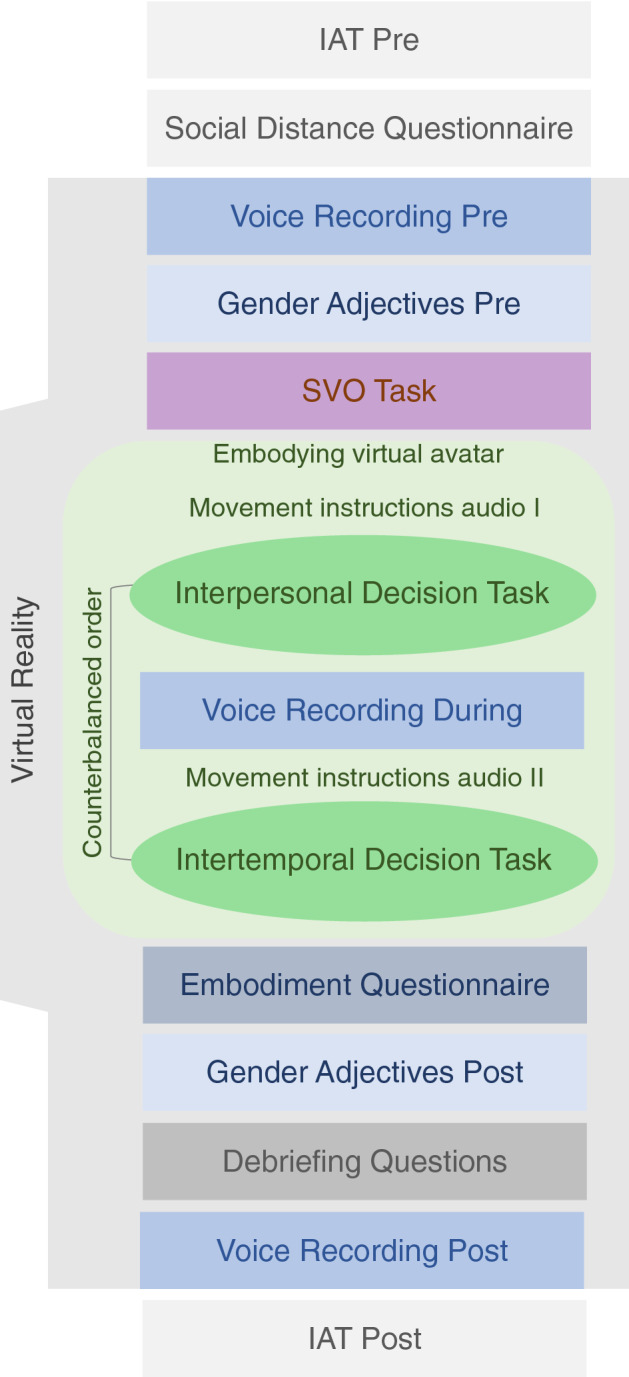
Experimental procedure. Overview of the experimental procedure. *IAT* implicit association test, *SVO* social value orientation.

The general procedure is illustrated in Fig. 2. After signing the informed consent form, participants completed a Self-Concept IAT on a desktop computer. They then filled out the social distance questionnaire, which was later used for the interpersonal discounting task. In the next step, participants put on the trackers and the head-mounted display, and were asked to complete the first voice recording tasks. Words were presented to them through the head-mounted display and participants read them out loud while being recorded. Subsequently, they performed the gender adjectives task, which was followed by a virtual implementation of the SVO task. After completion, the virtual SVO scene changed into a virtual room, where a typified “female” or “male” avatar was calibrated to the participant’s height by matching the physical trackers to the avatar’s limbs (Fig. 1B). After the instructed movements and exploring their virtual body in the mirror, participants completed the first decision task (counterbalanced order within group of either the interpersonal or intertemporal discounting task) on a virtual gesture-reactive screen monitor. A second embodiment induction audio followed, instructing participants to return to the virtually displayed computer, where they repeated the voice recording task. Next, they completed the second discounting task (either intertemporal or interpersonal, depending on the counterbalancing). For each trial in both tasks, participants chose one of the two options by placing their hand over it on the virtual touch screen and then confirming their decision via the continue-button. Following the last trial of the second task, the scene changed to black and participants filled out the embodiment questionnaire, which was implemented and presented virtually through the head-mounted display. After completion, they repeated the gender adjectives task, answered a few debriefing questions, and then completed the voice recording task a third time. Following completion of the voice recording task, participants were asked to take off the headset. Finally, a post virtual embodiment Self-Concept IAT, which was identical to the IAT administered prior to the virtual embodiment, was repeated on the desktop. The entire experiment lasted approximately 75 min. At the end, participants drew two random numbers from 1 to 54 by clicking on a button. The numbers corresponded to one trial in each decision task. Participants were paid according to their decision in that trial. We followed the procedure Soutschek and colleagues^21^ used to pay the participants. *Interpersonal decision task*: If participants’ decision was to share in the random trial, we gave them 7.50 Swiss francs in cash and either sent the other 7.50 Swiss francs to the other person via letter or went to the cafeteria to give the money to a stranger (in case the participant shared with someone of a social distance of 50 or 100). If participants chose to keep the money, they were paid the trial-specific amount in cash. *Intertemporal decision task*: If participants chose the now-option on the randomly selected trial, then they received the trial-specific amount in cash. If they chose the larger later option, we sent them 16 Swiss francs after the corresponding delay via letter mail.

### Data processing and analysis

Behavioral and questionnaire data were recorded in Unity and preprocessed in R (RStudio, Version 1.2.5033). The statistical analysis of the behavioral data was performed with Matlab R2016 (MathWorks, Natick, MA) and IBM SPSS Statistics 24.

For both the interpersonal and the intertemporal decision task, we computed hyperbolic discount functions that captured the discounted subjective value of generous and later rewards as a function of social distance and temporal delay. We fitted hyperbolic discount functions to the individual indifference values in these tasks. Each indifference value was defined as the point at which a participant chooses the generous vs. selfish reward or delayed vs. immediate reward with an equal probability of 50%. We computed indifference values separately with logistic regressions of choices on amount of the selfish reward option (interpersonal discounting task) or amount of the immediate reward option (intertemporal discounting task) separately for each social distance or temporal delay. The beta coefficients of each logistic regression allowed us to determine the selfish or immediate amount at which an individual was indifferent between choice options for a given social distance or delay. Finally, we fitted discount functions to these indifference values (using the function *lsqcurvefit* in Matlab) for each participant, assuming standard hyperbolic discount functions:

1 $${SV}_{social}= \frac{15 \,Swiss\, francs}{1+{k}_{social}\times Social \,distance}$$

2 $${SV}_{delay}= \frac{16 \,Swiss\, francs}{1+{k}_{delay}\times Delay}$$

where *SV*_social_ and *SV*_delay_ are the discounted values of the prosocial and delayed reward options, respectively, whereas *k*_social_ and *k*_delay_ represent participant-specific constants measuring the degree of social and intertemporal discounting. In social discounting, a larger *k*_social_ reflects more selfish decisions with increasing social distance, while in intertemporal discounting, a larger *k*_delay_ corresponds to more choices of the immediate reward option with longer delays. For the statistical analysis, the estimated parameters were log-transformed to normalize the skewed distributions of the parameters. We note that models of social discounting often include also a free intercept parameter which is sensitive to generosity towards close social others^21,25,36^. However, such a two-parameter model explained the observed data less well (median AIC = 11.4) than the one-parameter discount model (median AIC = 11.0). We therefore report only the results for the best-fitting one-parameter discount model. We also analyzed the behavior in interpersonal and intertemporal decision tasks with model-free mixed generalized linear models (MGLMs). For the interpersonal decision task, we regressed binary choices (0 = selfish option, 1 = prosocial option) on fixed-effects predictors for Sex (female vs. male), Group (experimental vs. control), Social distance, Selfish reward, and all interaction terms. As random effects, we modelled random slopes for Social distance, Selfish reward, and the interaction term in addition to participant-specific random intercepts. Analogously, in the intertemporal decision task, choices (0 = immediate option, 1 = delayed option) were predicted by fixed-effect predictors for Sex, Group, Reward magnitude, Delay, and all interaction terms. We also included participant-specific random intercepts and random slopes for Reward magnitude, Delay, and the interaction term.

IAT *d* scores were computed according to the procedure suggested in Greenwald et al.^37^ The sense of ownership over the virtual body was assessed by Embodiment Questions (Q1 + (1 – Q2) + Q3)/3, the sense of agency by Q4 and sense of embodying a body of the avatar’s gender by Q5. The voice recordings of each condition (pre, during, and post) were manually cut to the actual start and end of the task. Due to technical issues during the experimental procedure and noisy recordings, the voice recordings of two female and nine male participants had to be excluded. The recordings were processed with Praat^38,39^ in order to extract the mean and standard deviation of the pitch for each repetition. Data were tested for normality and then integrated accordingly. The Aligned Rank Transformation Analysis of Variance (ART ANOVA) is a non-parametric approach to factorial ANOVA that enables the analysis of main as well as interaction effects^40^. We calculated ART ANOVAs to evaluate the results of the questionnaires and gender adjectives because the data were not normally distributed.

## Results

### Discounting tasks

#### Interpersonal discounting task

**Figure 3:**
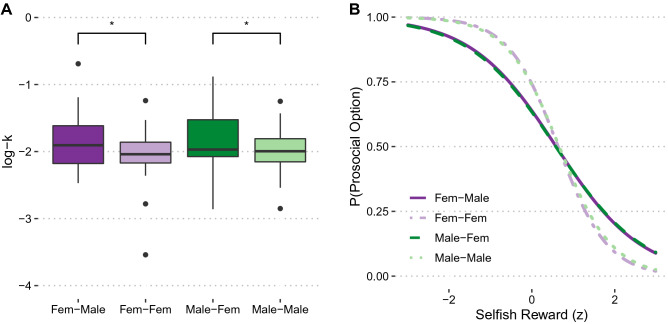
Interpersonal discounting task. (**A)** Log-transformed estimated discount factors *k*_social_ (log-k) by Sex and Group, median and interquartile ranges are displayed. A larger k reflects more selfish decisions with increasing social distance. ANOVA on log-ks revealed a significant difference for Group: Participants in different-gender avatars made less prosocial decisions with increasing distance than participants in same-gender avatars. (**B)** The probability of choosing a prosocial option as a function of *z*-transformed magnitude of selfish reward is displayed. MGLM on predictors Sex, Group, Social Distance, and Selfish Reward, revealed a significant Group × Selfish Reward magnitude interaction: Participants in different-gender avatars were less sensitive to the magnitude of the selfish reward than same-gender avatars.

To assess whether the body swap manipulation altered the generosity of female and male participants, we analyzed the log-transformed individually estimated discount factors *k*_social_ with a 2 (Sex: biological sex male or biological sex female) × 2 (Group: experimental (different-gender avatar) or control (same-gender avatar)) ANOVA. This analysis yielded a significant main effect of Group, *F*(1, 95) = 4.65, *p* = 0.03, *η*_p_^2^ = 0.047, indicating steeper social discounting in the different-gender (mean log-*k*_social_ = − 2.10) than in the same-gender avatar (mean log-*k*_social_ = − 1.85) group (Fig. 3A). Contrary to our hypothesis, however, we observed no evidence that the impact of the gender-swap manipulation differed between female and male participants, *F*(1, 95) = 0.32 , *p* = 0.57, *η*_p_^2^ = 0.003. Therefore, swapping into a different-gender (experimental group) rather than a same-gender (control group) avatar rendered behavior more selfish, independent of the participants’ or the avatar’s gender. This finding was further substantiated by the MGLM regressing binary choices (0 = selfish choice, 1 = prosocial choice) on predictors for Sex, Group, Social Distance, Selfish Reward, and the interaction effects (Table S1). Participants made more selfish choices with increasing social distance, *β* = − 3.14, *t*(77) = 13.13, *p* < 0.001, and with increasing magnitudes of the selfish reward option, *β* = – 1.62, *t*(94) = 7.63, *p* < 0.001. They were less sensitive to the magnitude of the selfish reward in the experimental groups compared with control groups, *β* = 0.66, *t*(85) = 2.22, *p* = 0.03 (Fig. 3B). Taken together, these findings suggest that choices are less generous in different-gender avatars than in same-gender avatars.

#### Intertemporal discounting task

**Figure 4:**
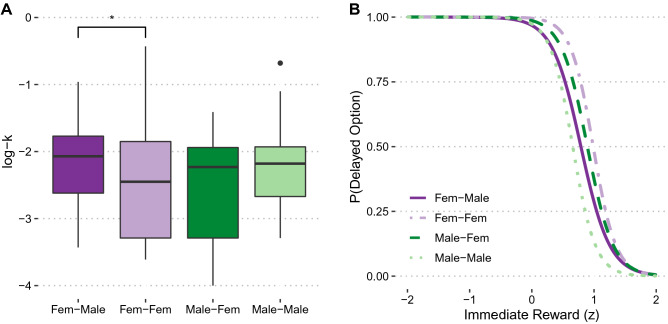
Intertemporal discounting task. (**A)** Log-transformed estimated discount factors *k*_delay_ (log-k) by Sex and Group, median and interquartile ranges are displayed. A larger k reflects more immediate choices with longer delay. ANOVA revealed a significant Sex × Group interaction, which was driven by female participants: they chose more smaller sooner rewards when they swapped into male bodies than into female bodies. (**B)** The probability of choosing a delayed option is displayed as a function of the magnitude of the immediate reward. MGLM revealed a significant Group × Reward magnitude interaction: Participants in different-gender avatars were less sensitive to the magnitude of the immediate reward than same-gender avatars as indicated by the less steep logistic curve.

A 2 (Sex) × 2 (Group) ANOVA on log-transformed hyperbolic discount parameters *k*_delay_ revealed no evidence for significant main effects of Sex or Group, both *F* < 1, both *p* > 0.58, *η*_p_^2^ = 0.047. However, we observed a significant Sex × Group interaction, *F*(1, 95) = 5.29, *p* = 0.01, *η*_p_^2^ = 0.067, suggesting that the gender-swap had dissociable effects for female and male participants: Female participants engaged in greater delay discounting (i.e., chose more smaller sooner rewards) when they swapped into male bodies than into female bodies, *t*(46) = 2.06, *p* = 0.04, *Cohen’s d* = 0.61, while we observed no significant effects for male participants, *t*(49) = 1.59, *p* = 0.12, *Cohen’s d* = 0.45 (Fig. 4A). This result is supported by the findings of a MGLM, revealing a significant Sex × Group interaction, *β* = − 1.65, *t*(73) = 2.03, *p* = 0.046 (Table S2). The effect of the gender swap on choices was stronger for female than male participants. Interestingly, we also observed a significant Group × Reward magnitude interaction, *β* = 0.96, *t*(115) = 2.11, *p* = 0.04, such that participants were less sensitive to immediate rewards (indicated by a less steep logistic curve describing the relationship between choices and reward magnitude) in the different-gender than in the same-gender group (Fig. 4B). Taken together, female participants chose smaller sooner rewards more often when making intertemporal choices in a male compared to a female avatar, and both were less sensitive to immediate rewards in the body of a different-gender avatar.

### IAT

According to the pre IAT *d* score, participants overall showed little (± 0.2) automatic association for their own gender before the experiment. To examine whether implicit self-association changed, we analyzed the *d *scores with a 2 (Time: pre and post) × 2 (Sex) × 2 (Group) repeated measures ANOVA, with Sex and Group as between-participant variables and IAT *d* scores as a within-participant variable. The results showed a significant effect for Time, *F*(1, 95) = 4.14, *p* < 0.05, *η*_p_^2^ = 0.042, indicating that the pre scores differed from the post scores, as well as a significant Time × Sex interaction, *F*(1, 95) = 7.09, *p* < 0.01, *η*_p_^2^ = 0.069, reflecting that *d* scores differed between female and male participants. However, the Time × Group interaction was not significant, *F*(1, 95) = 0.20, *p* = 0.66, *η*_p_^2^ = 0.002, nor was the Time × Sex × Group interaction, *F*(1, 95) = 0.07, *p* = 0.79, *η*_p_^2^ = 0.000. In contrast to our expectations, participants in the different-gender avatar thus did not seem to align implicit associations between their gender self-concept and gender of the avatar after the body swap. While the main effect of Sex is expected (i.e., that female participants show positive *d* scores, i.e., faster sort “self” with female, and male participants show negative *d* scores, i.e., faster sort “self” with male”), we assume that the main effect of Time merely reflects a repetition effect that commonly occurs when taking the IAT^41^. Mean IAT *d* scores are summarized in Figure S3.

### Gender adjectives

To assess explicit associations with gender, we asked participants to attribute gender adjectives to themselves. To verify the measures, we first tested whether the *pre* measure differed between female and male participants, irrespective of which avatar they later embodied. As expected, female participants initially rated themselves as more female, *Mdn* = 0.40, than male participants, *Mdn* = 0.48, Mann–Whitney U Test, *U* = 634, *p* < 0.001, *r* = − 0.48. To examine the difference between *pre* and *post* body swap VAS scores, we subtracted the *pre* from the *post* score and performed a 2 (Sex) × 2 (Group) ART ANOVA. There was no significant effect of Sex, *F*(1,95) = 3.41, *p* = 0.068, *η*_p_^2^ = 0.035, or Group, *F*(1, 95) = 1.69, *p* = 0.197, *η*_p_^2^ = 0.017, nor a Sex × Group interaction, *F*(1, 95) = 1.65, *p* = 0.2, *η*_p_^2^ = 0.017, indicating that participants did not change their explicit self-associations in response to the body swap.

### SVO task

We used the SVO task to assess baseline prosociality in the four groups of participants (Table 1). There was no significant evidence for unequal distributions of social value orientations among the four groups, Pearson’s Chi-squared test, *χ*^2^(9,99) = 16.87, *p* = 0.051, suggesting that baseline differences in prosociality should not explain group differences, even though the results almost reached significance. Furthermore, we note that the main effect of Group on social discount rates remains significant when we statistically controlled for baseline differences in SVO, *F*(1, 92) = 4.92, *p* = 0.03, *η*_p_^2^ = 0.051.

**Table 1:** SVO classification.

Group	SVO				Total
	Competitor	Individualist	Prosocial	None	
Fem-Male	0	6	18	2	26
Fem-Fem	1	4	13	7	25
Male-Fem	3	5	14	1	23
Male-Male	0	6	18	1	25
Total	4	21	63	11	99

*SVO* social value orientation by van Lange et al.^34^.

### Measure of embodiment

#### Sense of ownership

**Figure 5:**
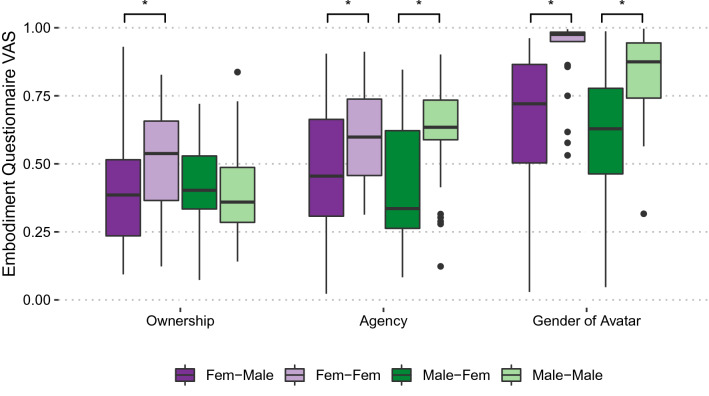
Embodiment questionnaire. Ownership, sense of agency and embodiment of avatar gender ratings. The data are separated by Sex and Group; median and interquartile ranges are displayed. *Ownership*: ART ANOVA revealed a significant Sex × Group interaction, driven by female participants, who experienced less ownership over their virtual bodies than male participants when swapping into different-gender avatars. *Agency*: ART ANOVA revealed significant effect for Group: Participants who swapped into a different-gender avatar reported lower agency than those who swapped into a same-gender avatar. *Gender of Avatar*: ART ANOVA revealed significant effect for Group: Participants who swapped into a different-gender avatar reported lower sense of the avatar’s gender than those who embodied a same-gender avatar; significant difference for Group in agency and sex of avatar.

Overall, the sense of ownership was low to moderate among all participants, *Mdn* = 0.43, *IQR* = 0.27. A 2 (Sex) × 2 (Group) ART ANOVA revealed no effect of Sex, *F*(1, 95) = 1.61, *p* = 0.21, *η*_p_^2^ = 0.017, nor Group *F*(1, 95) = 2.33, *p* = 0.131, *η*_p_^2^ = 0.024, but a significant interaction of Sex × Group *F*(1, 95) = 4.76, *p* < 0.05, *η*_p_^2^ = 0.048. Female participants experienced less ownership over their virtual bodies than male participants when swapping into different-gender avatars (Fig. 5).

#### Sense of agency

Participants reported moderate to high agency, *Mdn* = 0.59, *IQR* = 0.36. A 2 (Sex) × 2 (Group) ART ANOVA yielded no significant main effect for Sex, *F*(1, 95) = 0.17, *p* = 0.68, *η*_p_^2^ = 0.002. There was a significant effect of Group, *F*(1, 95) = 12.01, *p* < 0.01, *η*_p_^2^ = 0.113. Participants who swapped into a different-gender avatar reported a significantly lower sense of agency over their virtual body than participants who embodied the same-gender avatar (Fig. 5). The Sex × Group interaction was not significant, *F*(1, 95) = 0.70, *p* = 0.406, *η*_p_^2^ = 0.007.

#### Sense of embodying a body of the gender of the avatar

Participants strongly felt that they embodied a body of the avatar’s gender, *Mdn* = 0.81, *IQR* = 0.35. An ART ANOVA showed no significant main effect for Sex, *F*(1, 95) = 1.90, *p* = 0.171, *η*_p_^2^ = 0.020, but did for Group, *F*(1, 95) = 29, *p* < 0.001, *η*_p_^2^ = 0.235. Participants of both sexes in the experimental group reported lower embodiment of the different-gender avatar than the control participants who embodied a same-gender avatar (Fig. 5). The Sex × Group interaction was not significant, *F*(1, 95) = 0.35, *p* = 0.555, *η*_p_^2^ = 0.004.

### Voice recordings

We analyzed the recordings with 3 (Time: pre, post, and during) × 2 (Sex) × 2 (Group) mixed and repeated measures ANOVA, with Sex and Group as between-participant variables and Pitch (*F0*) as a within-participant variable. The assumptions of sphericity were violated as indicated by Mauchly’s Test, *χ*^2^(19.82) = 0.79, *p* < 0.001; therefore, degrees of freedom were corrected using Huynh–Feldt estimates of sphericity, *ε* = 0.84. The results showed no main effect for Time, *F*(1.68, 140.98) = 2.65, *p* = 0.083, *η*_p_^2^ = 0.031, and revealed no Time × Group interaction, *F*(1.68, 140.98) = 0.25, *p* = 0.744, *η*_p_^2^ = 0.003, Time × Sex interaction, *F*(1.68, 140.98) = 0.32, *p* = 0.688, *η*_p_^2^ = 0.004, nor a Time × Sex × Group interaction, *F*(1.68,1 40.98) = 0.18, *p* = 0.794, *η*_p_^2^ = 0.002. As expected, the mean pitch frequency was higher in female compared to male participants (as reflected by the between-participant effect of Sex, *F*(1,84) = 294.30, *p* < 0.001, *η*_p_^2^ = 0.778). However, participants did not seem to differentially change their voice after swapping into the body of a different-gender or a same-gender avatar, as we found no significant between-participant effect for Group *F*(1, 84) = 0.08, *p* = 0.755, *η*_p_^2^ = 0.001, nor a Sex × Group interaction, *F*(1,84) = 0.33, *p* = 0.570, *η*_p_^2^ = 0.004.

### Debriefing questions

#### Influence of the virtual body’s identity

**Figure 6:**
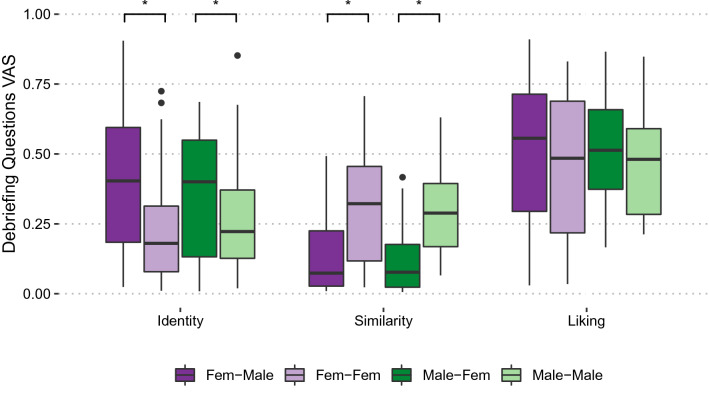
Debriefing questions. Debriefing question ratings. The data are separated by Sex and Group; median and interquartile ranges are displayed. *Identity*: Explorative ART ANOVA revealed a significant effect for Group, i.e., participants who swapped into a different-gender avatar felt more like their decisions were influenced by the virtual identity than those who swapped into a same-gender avatar. *Similarity*: Explorative ART ANOVA revealed a significant effect for Group, participants who swapped into different-gender avatars reported lower perceived similarity to their physical appearance than those who swapped into a same-gender avatar. *Liking*: No significant effects were found.

The agreement on whether the virtual avatar’s identity influenced participants’ decisions on the tasks was generally low, *Mdn* = 0.25, *IQR* = 0.42. An explorative 2 (Sex) × 2 (Group) ART ANOVA did not reveal a significant main effect for Sex, *F*(1, 95) = 0.01, *p* = 0.926, *η*_p_^2^ = 0.001, but did for Group, *F*(1, 95) = 6.12, *p* < 0.05, *η*_p_^2^ = 0.061. Participants who swapped into a different-gender avatar felt more like their decisions were influenced by the virtual body than those who swapped into a same-gender avatar (*Fig. **6**)*. The Sex × Group interaction was not significant, *F*(1, 95) = 1.20, *p* = 0.275, *η*_p_^2^ = 0.013.

#### Perceived similarity to physical body

On average, participants did not seem to perceive the virtual body to look similar to their physical body, *Mdn* = 0.17, *IQR* = 0.28. Again, we conducted an explorative 2 (Sex) × 2 (Group) ART ANOVA that revealed no significant main effect for Sex, *F*(1, 95) = 0.17, *p* = 0.681, *η*_p_^2^ = 0.002, but did for Group, *F*(1, 95) = 32.12, *p* < 0.001, *η*_p_^2^ = 0.253. Participants who swapped into different-gender avatars reported lower perceived similarity to their physical appearance than participants who swapped into a same-gender avatar (Fig. 6). The Sex × Group interaction was not significant, *F*(1, 95) = 0.47, *p* = 0.493, *η*_p_^2^ = 0.005.

#### Liking of the virtual body

Participants were neutral about the virtual avatar’s looks (*Fig. **6*), *Mdn* = 0.50, *IQR* = 0.37. An explorative 2 (Sex) × 2 (Group) ART ANOVA did not indicate an effect for Sex, *F*(1,95) = 0.02, *p* = 0.902, *η*_*p*_^*2*^ = 0.0002 or Group, *F*(1, 95) = 0.936, *p* = 0.336, *η*_*p*_^*2*^ = 0.010, nor a Sex × Group interaction, *F*(1, 95) = 0.06, *p* = 0.815, *η*_*p*_^*2*^ = 0.001.

## Discussion

The current study investigated the influence of a virtual gender swap on interpersonal and intertemporal discounting tasks, as well as on implicit and explicit identification with gendered (or gender-typical) traits. We expected that embodiment of a female avatar would stimulate generosity, while embodiment of a male avatar would increase selfish choices on the interpersonal discounting task, independent of participants’ own biological sex. Initially having included the intertemporal discounting task as a control measure, in line with Soutschek et al.^21^, we expected the virtual gender swap to exert no discernible influence on delay discounting. The results suggest three main findings: *First*, regardless of their biological sex, participants made more selfish choices in the interpersonal discounting task when they embodied a different-gender avatar (i.e., female participants in a male avatar or vice versa). *Second,* women in different-gender avatars in particular chose options providing smaller but sooner rewards more often in the intertemporal discounting task, thereby demonstrating increased delay discounting. *Third*, there was no evidence that the different-gender swap illusion in our setup altered either implicit or explicit identification with gendered (or gender-typical) traits.

### People make more selfish choices during gender swap

In line with the Proteus effect^7^, the phenomenon in which embodiment of virtual surrogates can elicit behaviors effects that reflect the stereotyped identity characteristics of the embodied avatar, we hypothesized that participants would adjust their decision-making on the two discounting tasks according to the gender-stereotypical characteristics of the avatar’s gender. Specifically, we expected participants embodying a female avatar to more often select generous options, while those embodying a male avatar were presumed to engage in more selfish decision-making. Contrary to this hypothesis, however, we did not find a main or interaction effect of the avatar’s gender on the interpersonal discounting task. Although a recent meta-analysis reported generally reliable small to medium effect sizes for the Proteus effect^42^, other studies reported no or even opposite effects. For example, Sherrick et al.^43^ found that participants who adopted the role of a male (versus female) protagonist in an interactive fictional story were less likely to select masculine behaviors, but would instead display behaviors stereotypically associated with being female. However, studies focusing on the Proteus effect often differ in their methodological implementations, ranging from the type of virtual embodiment (i.e., immersive VR or two-dimensional desktop avatars) to the choice of behavioral tasks. Furthermore, many potentially moderating factors, such as the strength of belief in stereotypes or identification with the surrogate avatar could effectively alter behavior as well. Therefore, further research examining user-avatar dynamics within the context of different experimental paradigms will help in providing more detailed insight and further credence to the specific particulars of the Proteus effect.

Despite the absence of the hypothesized main effect of the avatar’s gender on interpersonal discounting in our study, we did find an effect of different-gender avatar on generosity. Specifically, both female and male participants chose more selfish options when they embodied a different-gender avatar. In line with this, the same participants were less sensitive to the immediate amount they could receive in intertemporal decisions, which converges with the increased selfish behavior: We assume that the less selfish behavior in the same-gender avatar groups lead to a stronger focus on the amount when deciding to share versus not to share. Though the current study did not experimentally manipulate or individually correlate potential modulators, it is possible that the reduced sense of agency or increased sense of dissimilarity experienced by participants in the different-gender avatar could have elicited more selfish decisions.

Although we can currently only speculate about the underlying mechanisms, the reduced sense of agency reported by the different-gender avatar participants could have constituted a driving factor for choice behaviors on the interpersonal discounting task. The sense of being in control of our own actions implies an inherent sense of personal responsibility that rests on voluntary choice, which may in turn promote moral preferences. Previous studies have demonstrated that both a stronger sense of ownership and agency are associated with strengthened moral identity^44,45^, while performance of immoral actions or moral disengagement are conversely associated with lower perceived agency^46,47^. Furthermore, several studies have examined the link between the sense of responsibility and sense of agency retrospectively^48–50^. Metcalfe and Greene^49^ suggest that participants are very good at judging whether their agency is high or not, and subsequently assess their performance in a task accordingly: They found that the sense of responsibility for both bad and good outcomes in the task was decreased when the sense of agency was low and increased when it was high. Therefore, the reduced generosity on the interpersonal discounting task of participants in the different-gender avatar condition could potentially be explained by diminished personal responsibility that accompanied the decreased sense of agency reported in these groups. However, one could also expect an effect in the other direction, where participants who behaved more selfishly in the interpersonal task may have subsequently experienced a diminished sense of agency as a coping method to distance themselves from their behavior, which would be an interesting question for future research.

In addition to the diminished sense of agency, participants in the different-gender avatar also reported larger perceived dissimilarity to the virtual body, which could further account for the reduced generosity observed. Previous research has proposed that the so-called “online disinhibition effect”^51^, which maintains that use of a pseudonym or an avatar, rather than one’s real name or picture, in online (or digital) environments increases perceived anonymity. This perceived anonymity in turn may create a compartmentalized “online self”, which can perceivably exist in an imaginative online dimension that stands separate from the offline (in-person) self. Furthermore, the behavior of this online identity might differ quite strongly from a person’s offline behavior, including decreases in personal responsibility and moral cognitive processing. According to Suler^51^, acting under the guise of a virtual identity can promote a dissociative anonymity that encourages individuals to separate their digital behavior from their in-person characteristics, thereby facilitating a disinhibition of behaviors that would normally be more suppressed by the in-person self. Therefore, participants who embodied the perceived dissimilarity between the virtual different-gender avatar and the physical own body may have facilitated an increased sense of dissociative anonymity that could have concomitantly reduced the sense of personal responsibility (i.e., averting it to the virtual different-gender body), thereby providing a guise to engage in more selfish behaviors. It could further be speculated that this form of altered self-identification could have additionally temporarily modulated the perceived degree of closeness and the relations towards the specific individuals that participants reported on their respective social distance lists. Therefore, the interpersonal relations to these people could have shifted, which might have encouraged greater social discounting behaviors.

Lastly, it should be noted that we did not replicate the previously reported differences between men and women in the interpersonal discounting task, even when they embodied their own gender^21^, which could suggest that participants might use different strategies for this task in embodied VR, as compared to a non-mediated environment. However, our control SVO measure also did not reveal gender differences in our sample’s baseline prosociality. Accordingly, it is reasonable to expect that men and women of same-gender avatars in our study would similarly not differ in their social discounting behaviors.

### Women in male avatars chose smaller sooner options more often

Although our initial hypothesis stated no gender differences in intertemporal decision-making, it should be noted that the empirical findings surrounding gender differences on this task are somewhat mixed, as another study found that men rather displayed stronger preferences for immediate (i.e., smaller sooner) over larger later rewards than women^52^. Our findings evince partial support for this alternate view; specifically, we found that women tended to prefer immediate over delayed rewards when embodying a male avatar, although no such differences were found in men. While gender differences have been described in potentially related psychological constructs^20^, such as impulsivity^53^, only very few studies have looked at the effect of sex in the intertemporal discounting paradigm. Contrary to Soutschek et al.^21^, who found no delay discounting differences between men and women, Dittrich and Leipold^52^ reported that female participants were more patient, whereas male participants tended to choose the immediate payment more often. This held true especially if the interest rate of the larger later option was neither too high nor too low. Our results showing that women in male avatars more often chose smaller sooner rewards are therefore in line with previous findings of gender-specific effects on this task. As predicted, women in male virtual bodies in our study therefore seemed to follow the Proteus effect, whereby they assimilated stereotypically male characteristics (e.g., impulsivity) to engage in greater delay discounting behaviors. In line with our interpretations of the interpersonal discounting task, we here similarly assume that the lower sense of agency and ownership in the women embodying a male avatar could have elicited a decreased sense of responsibility^44^. However, it is unclear why we did not observe men in a female avatar to choose larger later rewards more frequently.

Furthermore, although we did not administer any baseline control measures to account for potential gender differences in the intertemporal discounting task, it is plausible that the men and women in our sample, similarly to the SVO task, may not have differed in potentially contributing moderating psychological constructs, such as self-control. Therefore, the observed effect specific to women in male avatars may hint at a particularly instrumental effect of virtual embodiment on delay discounting in this group. Lastly, it is worth noting that many participants in the current study were students recruited from the Economics Department. Research has demonstrated that economics students can show systematically different behaviors than non-economic students, such as demonstrating strong self-interest and acting more selfishly^54–56^. Although intertemporal discounting cannot directly be compared to selfish decision-making, it is nevertheless possible that economics students possessed either prior knowledge of the intertemporal discounting task or exhibit moderating characteristics that could have substantially influenced their choices.

### Gender swap does not alter identification with gendered traits

Previous research suggested a change in implicit and explicit identification with gendered (or gender-typical) traits after a gender swap illusion^15^. Furthermore, Mello et al.^14^ reported that swapping into a virtual body of the opposite gender led heterosexual women and men to rate intimate touch as more pleasant and erogenous when it came from a toucher of the same sex, suggesting that gender-specific cognitive representations of the (sexual) self in heterosexual participants changed during the swap. In the current setup, however, we did not find any effect of the gender swap on either explicit traits (as measured using self-attribution of gender stereotyped adjectives) or implicit gender-identification (as measured by the IAT). Furthermore, results also did not provide evidence for a change in participants’ voices, which stands in contrast to previous studies that had demonstrated voice changes following body swaps^10,11^. It is important to note that the absence of an effect in our study cannot simply be explained by a lack of illusory embodiment. First, although ownership ratings were rather low, they were comparable to those reported by Peck et al.^5^, who observed a Proteus effect in female participants performing a working memory task in same-gender versus different-gender avatars. Second, the agency ratings in our sample were medium to high. Third, and most importantly, participants strongly agreed that they felt embodied in the avatar’s gender. This suggests that, indeed, participants transiently embodied both the different-gender and the same-gender avatar. We therefore believe that the lack of effect on implicit and explicit measures of gender identity might rather be linked to differences in our design compared to previous studies.

To the best of our knowledge, there is only one study that directly assessed the effects of a gender swap illusion on implicit and explicit gender identity^15^. Using an explicit gender adjectives task and an auditory version of the IAT, the authors reported a more balanced identification with both genders and less gender-stereotypical beliefs about one’s own personality characteristics. However, a few important differences should be considered between this recent study and our study. First, the setup used by Tacikowski et al.^15^ consisted of a video-based gender swap paradigm, in which participants viewed the physical body of another person through the head-mounted display. Furthermore, they additionally employed visuotactile stimulation (i.e., synchronicity between the felt touch on the own body and seen touch on the video-based virtual body) to strengthen the embodiment illusion. In contrast, our setup was based on a computer-generated gender swap (virtual avatar), where the illusion was induced by visuomotor synchrony (real-time full body tracking) and enhanced with audio instructions. Virtual avatars do not (yet) emulate physical bodies in such detail that they could be comparable to physical bodies; therefore, it is plausible that the more naturalistic video-based setup used by Tacikowski et al.^15^ could have enhanced the perceived effects of the gender swap. Second, their design was a within-subject design, in which participants experienced both genders, each stimulated both synchronously (to induce embodiment) and asynchronously (to prevent embodiment). While not empirically confirmed, such repeated conditions could have made participants more aware of the study’s purpose, which may then have facilitated the change in explicit identification with gendered (or gender-typical) traits. This potential issue is the reason we did not use a within participant design. Third, while design choices would not necessarily justify the implicit measure changes in the study by Tacikowski et al.^15^, it is important to note that they measured the IAT during rather than after the gender swap, which might be a reason for the different findings. Although some studies have demonstrated sustained changes in implicit biases on the IAT^16^, the few studies that have examined the longevity of such changes had employed IAT measures focused on social cognition, rather than self-related cognition.

Finally, we did not find any effects of gender swap on voice pitch. Although previous studies have shown such effects, methodological design choices here too differed in that they concurrently presented auditory vocal cues in multimodal settings^30^. In particular, participants heard a voice (the avatar’s) while synchronously seeing the avatar’s lips (in a virtual mirror) move in correspondence with the spoken utterances (lip sync). Changes in the participants’ vocal pitch were associated with visuomotor synchrony of the body together with vocal vibrotactile cues^30^ and with visuomotor synchrony of the body and a modulation of participants’ own voice^11^, but not with non-vocal visuotactile stimulation to the body^11^. In contrast, no concurrent auditory-vocal cues were presented in our study. Our findings thus suggest that embodiment of a stereotyped body might not be enough to result in pitch shifts, but that additional multisensory stimulation using vocal cues seem necessary. Further research is needed to fully investigate the link between vocal cues and illusory embodiment, in particular, whether a subjective link between the embodied avatar and the heard voice is necessary to result in pitch shifts, and whether the auditory feedback is sufficient or the audiovisual lip sync is important.

### Limitations and outlook

There are some important limitations that should be considered. In line with Peck et al.^5,6^, the current study simplifies gender to a binary concept that does not necessarily correspond to the gender identity and expression on a non-binary continuum in reality. Additionally, participants embodied generic, typified avatars during the swap and did not experience a particularly strong embodiment illusion. Personalized avatars and a high degree of immersion (i.e., matching the shape of the avatar’s 3D body to the participant’s physical body) that increase the similarity between the virtual and physical body could enhance the sense of ownership and agency over the virtual body^57,58^. Even though non-personalized male and female avatars have previously elicited the Proteus effect^5,6^, future studies incorporating advanced technological possibilities could examine the influence of personalized avatars in comparison to generic ones. Increasing similarity and immersion between the avatar and the physical body could potentially lead to increased liking of the avatar’s appearance, and therefore to a strengthened embodiment illusion. On the flip side, we hypothesized that the sense of dissimilarity between the own and virtual body could have reduced participants’ sense of agency and therefore influenced decision-making. We argued that embodying the avatar of a different gender enhanced this sense of dissimilarity; however, future research should examine whether this effect is specific to gender differences, or whether embodying a virtual body that differs in other characteristics (e.g., age) could similarly affect decision-making.

Although we used the SVO as a measure of prosociality, we did not administer a non-VR based baseline measurement of participants’ behavior in the interpersonal and intertemporal decision tasks to which we could have compared their behavior during the virtual body swap. Additionally, it should be noted that the interpersonal and intertemporal discounting tasks were both administered during the virtual embodiment session, while the implicit and explicit gender identification measures were assessed prior to and following the VR session, rather than during the virtual embodiment. Although studies have demonstrated effects of VR embodiment over an extended time (e.g., one week^16^) following virtual embodiment, future studies assessing such tasks both during the virtual embodiment session, as well as assessment of the longevity of such effects, would be very informative.

## Conclusion

The present research provides a first investigation of how a virtual gender swap affects social and delay discounting in women and men. Our results demonstrate that both women and men tend to engage in more selfish behaviors when virtually swapping genders, and that women embodying male avatars engage in more delay discounting. Together, the current study suggests that a virtual gender swap can selectively and differentially affect the choice behavior of women and men.

Technological advancements have increased the availability of virtual online worlds. While two-dimensional interactive spaces have been commonplace in our daily lives for several decades, the recent proliferation of consumer-level head-mounted displays and VR systems are paving the way for the significantly prominent role of immersive VR in our interpersonal lives. As an increasing number of individuals look to VR as a novel tool for advancing user experiences in health, education, and entertainment, understanding how embodied virtual identity manipulations impact behavior is of particular importance.

## Supplementary Information

Supplementary Information.

## Data Availability

The data is available on the Open Science Framework, see https://osf.io/2czqk/.
