# Supplementary material for: Effects of a virtual gender swap on social and temporal decision-making
Source: Sci Rep. 2021 Jul 28;11:15376. doi: 10.1038/s41598-021-94869-z (PMC8319130; doi:10.1038/s41598-021-94869-z)
Supplement: Supplementary file 1 — Supplementary Information. [file 41598_2021_94869_MOESM1_ESM.pdf]

Supplementary Information

Effects of a Virtual Gender Swap on Social and Temporal Decision-Making

Elena Bolt, Jasmine T. Ho, Marte Roel Lesur, Alexander Soutschek,
Philippe N. Tobler, Bigna Lenggenhager

Correspondence to elena.bolt@hotmail.com
or bigna.lenggenhager@gmail.com

This document includes

- Transcripts of instructed moving audios I and II
- Supplementary Tables S1 and S2
- Supplementary Figure S3

Audio I: Transcript of movement instructions (5 minutes)

Welcome in your avatar! Your first task starts now. Please follow my instructions. First, look at your two forearms by raising them. Please do not rotate your wrists while doing this.

Next, look down and look at your body.

Move your feet.

Turn to the left towards the small mirror and walk towards the table. Please do not cross the blue mark on the floor. Place your hands on the table surface.

Now look at your face in the mirror.

Turn your head to the right and to the left.

Now wave to yourself with your right arm.

And wave with your left arm.

Take two steps backwards and take some time to look around the room.

Towards the door on the left side you will find a large wall mirror. Please stand in front of this mirror so that you can look at your body. Make sure that your feet are behind the red mark on the floor.

Walk a few steps backwards and forwards between the red and orange marks. Watch yourself as you do this. You can move your arms while doing so.

Turn towards the door and look at your body in the mirror from the side.

Repeat the same for the other half of your body.

Now take a few seconds to look at yourself in the mirror.

Thank you. The task is now finished. For the next task, please go back to the table and stand with your feet behind the blue marker.

Audio II: Transcript of movement instructions (2 minutes and 40 seconds)

Please look in the mirror above the computer. Look alternately at the ceiling and straight ahead again. While doing so, observe your movements in the mirror.

Take a step back and wave to yourself in the mirror with both hands at the same time.

Please stand again behind the red mark in front of the large mirror on the wall.

First stretch out your right leg without bending your knee. And then stretch out your left leg. Repeat these movements alternately for a few seconds.

Turn to the door and look at your body in the mirror from the side. Repeat the leg movements you did before alternately.

Repeat the same for the other half of your body.

Now walk a few steps backward and forward again between the red and orange marks. Watch yourself as you do this. You can move your arms to do this as you wish.

Thank you. The task is now finished. For the next task, I ask you to go back to the table and stand with your feet behind the blue marker.

Table S1. Results of generalized mixed linear model on binary choices (0 = selfish option, 1 = prosocial option) in the interpersonal decision task. Standard errors are indicated in brackets.

	beta	t-value	df	p-value
Intercept	1.05 (0.37)	2.87	88	0.005
Reward magnitude	-1.62 (0.21)	7.73	90	<0.001
Distance	-3.14 (0.24)	13.13	77	<0.001
Sex	0.01 (0.37)	0.03	77	0.98
Group	-0.49 (0.52)	0.94	76	0.35
Reward magnitude × Distance	-0.11 (0.16)	0.70	81	0.49
Sex × Reward magnitude	-0.05 (0.21)	0.24	90	0.81
Sex × Distance	0.02 (0.24)	0.07	77	0.95
Group × Reward magnitude	0.66 (0.30)	2.22	85	0.03
Group × Distance	0.41 (0.34)	1.21	72	0.23
Sex × Group	0.00 (0.52)	0.00	76	1.00
Sex × Reward magnitude × Distance	-0.28 (0.16)	1.70	81	0.09
Group × Reward magnitude × Distance	-0.22 (0.23)	0.96	76	0.34
Sex × Group × Reward magnitude	0.04 (0.30)	0.13	85	0.90
Sex × Group × Distance	-0.10 (0.34)	0.29	72	0.77
Sex × Group × Reward magnitude × Distance	0.17 (0.23)	0.75	76	0.45

Table S2. Results of generalized mixed linear model on binary choices (0 = immediate option, 1 = delayed option) in the intertemporal decision task. Standard errors are indicated in brackets.

	beta	t-value	df	p-value
Intercept	4.53 (0.58)	7.84	119	<0.001
Reward magnitude	-5.42 (0.33)	16.27	130	<0.001
Delay	-1.79 (0.22)	-8.21	149	<0.001
Sex	1.02 (0.58)	1.77	75	0.08
Group	-0.38 (0.81)	0.47	73	0.64
Reward magnitude × Delay	0.33 (0.18)	1.84	114	0.07
Sex × Reward magnitude	-0.09 (0.33)	0.27	130	0.79
Sex × Delay	-0.07 (0.22)	0.33	149	0.74
Group × Reward magnitude	0.96 (0.45)	2.11	115	0.04
Group × Delay	-0.33 (0.31)	1.09	152	0.28
Sex × Group	-1.65 (0.81)	2.03	73	0.046
Sex × Reward magnitude × Delay	0.12 (0.25)	1.70	114	0.51
Group × Reward magnitude × Delay	0.42 (0.25)	1.70	106	0.09
Sex × Group × Reward magnitude	0.33 (0.45)	0.72	115	0.47
Sex × Group × Delay	0.12 (0.31)	0.39	152	0.69
Sex × Group × Reward magnitude × Delay	-0.32 (0.25)	1.28	107	0.20

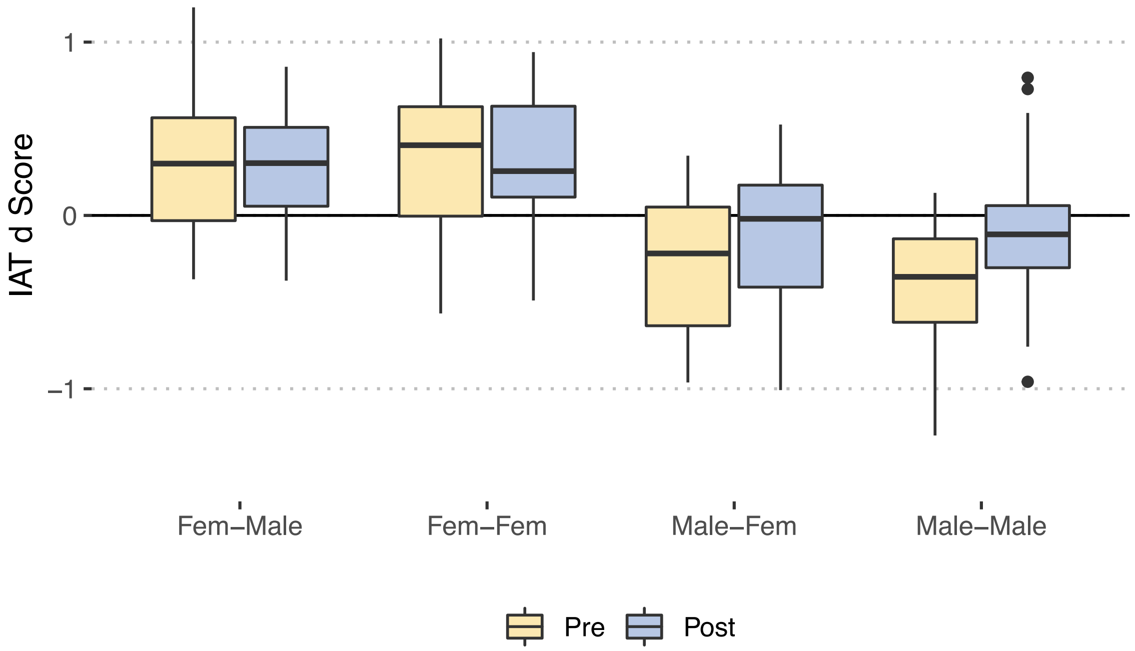

Figure S3, Self-Concept IAT. IAT *d* scores before (pre) and after (post) body swap by Sex and Group, median and interquartile ranges are displayed. IAT, Implicit Association Test. ANOVA revealed a significant effect of Sex, indicating that *d* scores differ between female and male participants (i.e., female showed positive scores, representing faster sorting of "female" with self" and male participants showed negative scores, representing faster sorting of "male" with "self"), which supports the self-concept theory underlying this IAT version. We also found a significant effect for Time, which merely reflects a repetition effect on the task. Significance is not displayed in the plot.
